# *Antrodia cinnamomea* alleviates cisplatin-induced hepatotoxicity and enhances chemo-sensitivity of line-1 lung carcinoma xenografted in BALB/cByJ mice

**DOI:** 10.18632/oncotarget.4348

**Published:** 2015-06-27

**Authors:** Tse-Hung Huang, Yi-Han Chiu, Yi-Lin Chan, Hang Wang, Tsung-Lin Li, Chien-Yin Liu, Cheng-Ta Yang, Tzung-Yan Lee, Jyh-Sheng You, Kuang-Hung Hsu, Chang-Jer Wu

**Affiliations:** ^1^ Department of Traditional Chinese Medicine, Chang Gung Memorial Hospital, Keelung, Taiwan; ^2^ Graduate Institute of Clinical Medicine Sciences, Chang Gung University, Taoyuan, Taiwan; ^3^ Department of Food Science, National Taiwan Ocean University, Keelung, Taiwan; ^4^ Department of Life Science, Chinese Culture University, Taipei, Taiwan; ^5^ Institute of Biomedical Nutrition, Hung Kuang University, Taichung, Taiwan; ^6^ Genomics Research Center, Academia Sinica, Taipei, Taiwan; ^7^ Department of Thoracic Medicine, Chang Gung Memorial Hospital and Chang Gung University, Taoyuan, Taiwan; ^8^ Department of Respiratory Care, Chang Gung University, Taoyuan, Taiwan; ^9^ Graduate Institute of Traditional Chinese Medicine, College of Medicine, Chang Gung University, Taoyuan, Taiwan; ^10^ School of Traditional Chinese Medicine, Chang Gung University, Taoyuan, Taiwan; ^11^ Laboratory for Epidemiology, Department and graduate institute of health care management, Chang Gung University, Taoyuan, Taiwan; ^12^ Center of Excellence for the Oceans, National Taiwan Ocean University, Keelung, Taiwan

**Keywords:** antrodia cinnamomea, cisplatin, hepatotoxicity, lung cancer

## Abstract

Whereas cisplatin (cis-diamminedichloroplatinum II) is a first-line medicine to treat solid cancerous tumors, it often causes serious side effects. New medicines that have an equivalent or even better therapeutic effect but with free or less side effects than cisplatin are highly anticipated in cancer therapy. Recent reports revealed that *Antrodia cinnamomea (AC)* possesses hepatoprotective activity in addition to anticancer. In this study, we wanted to know whether *AC* enhances chemo-sensitivity of cisplatin and/or alleviates cisplatin-induced hepatotoxicity, as well as the underlying mechanisms thereof. Our results indicated that *AC* inhibited proliferation of line-1 lung carcinoma cells and rescued hepatic HepG2 cells from cisplatin-induced cell death *in vitro*. The fact is that *AC* and cisplatin synergized to constrain growth of line-1 lung carcinoma cells in BALB/cByJ mice. Quantitative real-time PCR further revealed that *AC* promoted expression of apoptosis-related genes, while it decreased expression of NF-κB and VEGF in tumor tissues. In liver, *AC* reduced cisplatin-induced liver dysfunctions, liver inflammation and hepatic apoptosis in addition to body weight restoration. In summary, *AC* is able to increase cisplatin efficacy by triggering expression of apoptosis-related genes in line-1 lung cancer cells as well as to protect liver from tissue damage by avoiding cisplatin-induced hepatic inflammation and cell death.

## INTRODUCTION

Lung cancer known for its high relapse and low cure rate is the leading cancer, accounting for 19.5% of all cancer death [1], wherein the non-small-cell lung cancer (NSCLC) takes up 75–80% with a five-year survival rate of 10–15%. The non-resectable lung cancer (∼80% of NSCLC) is even notorious for its bad prognosis. The mean survival time of 9–23 months is contingent upon the epidermal growth factor receptor (EGFR) mutations and with/without EGFR-tyrosine kinase inhibitors (TKI) treatment [2, 3]. Though a subset of patients with specific EGFR mutations benefits from the EGFR targeted therapy [4, 5], for most patients the disease one way or the other advances as result of acquiring drug resistance [6].

Surgery, chemotherapy and target therapy are mainstream cancer treatments. In chemotherapy, the platinum-based chemotherapy remains one of the major choices for NSCLC. Cisplatin, a platinum-containing compound, is effective on a variety of solid tumors, such as lung, ovarian, malignant pleural mesothelioma and head and neck cancers [7, 8]. The fact is that cisplatin-based adjuvant chemotherapy has been estimated with high overall survival and/or disease-free survival rate in five large-scale clinical trials over 4584 fully NSCLC resected patients [9]. Both disease-free and overall survival rates of the NSCLC resected patients were increased by co-administration of cisplatin and vinorelbine [10]. Pepe *et al*. [11] suggested that elderly surgical NSCLC-removal patients treated with vinorelbine along with less total cisplatin chemotherapy would improve overall prognosis. Therefore, the cisplatin-based chemotherapy should not be completely withheld from elderly patients. Although younger patients could tolerate more aggressive cisplatin chemotherapy, while the one-year survival rate was not significantly increased by such an aggressive treatment [12]. While the therapeutic efficacy of cisplatin can be improved through dose expansion, serious non-specific cytotoxicity such as nephrotoxicity [13], ototoxicity [14] and hepatotoxicity [15] often arise. Nephrotoxicity is the most common dose-limiting cytoxicity, which looms slowly but predictably upon repeated exposure. Cisplatin accumulates in kidney higher than in other organs due to relatively dense mediated transport system in kidney, where from about one third of patients complicate with the cisplatin-induced nephrotoxicity [13, 16]. Hepatotoxicity is also very common in patients under high-dose cisplatin treatment [15]. Poor outcomes such as low survival rate, bad tumor responses, deteriorated life quality, and ill toxicity in advanced NSCLC patients underline urgent needs for better treatments [17].

Oxidative stress has been implicated in the pathogenesis of cisplatin-induced nephrotoxicity. Oxidative stress is also known positively correlated to reactive oxygen species (ROS) but negatively to antioxidant defense systems, for example, antioxidant enzymes and non-enzymatic molecule glutathione (GSH) [18, 19]. Liver, the key detoxification organ, is vulnerable to oxidative stress. Because the pathogenicity of the cisplatin-induced hepatotoxicity has not yet been clearly elucidated, the development of proper adjuvant treatments to prevent or alleviate the cisplatin-induced hepatopathy is hampered. Given that the cisplatin-induced liver injury is strongly associated with free radicals, a new strategy combing both cisplatin and antioxidant might be worthwhile to assess for reduction of the toxic side effects in liver [20, 21].

*Antrodia cinnamomea (AC)*, the genus Antrodia (Polyporaceae), is a photophobic parasitic fungus growing in inner cavities of *Cinnamomum kanehirai*, which is a Taiwan endemic species [22]. Taiwanese have a long history to use *AC* as a medicine for several body disorders, such as diarrhea, intoxication, hypertension, stomachache, inflammation, etc [23]. Recent studies further revealed that *AC* has some hepatoprotective effects, for example, inhibition of viral antigen activity of hepatitis viruses [24], reduction of oxidative stress of alcohol-induced liver diseases, and counteraction of liver fibrosis of the TAA-induced liver injury [25]. *AC* was also reported able to protect liver cells from free radical-induced oxidative stresses through the Nrf-2 activation and up-regulation of the MAP kinase-mediated antioxidant genes [26, 27]. Moreover, *AC* was reported able to inhibit proliferation of head and neck cancer cells [28], migration of leukemia cells [29, 30], as well as help characterize cancer stem cells of hepatocellular carcinoma [31].

In this report, we aimed to interpret the mechanisms of the *AC* protection effects on the cisplatin-induced oxidative stress and liver injury, as well as the inhibition effect of *AC* in lung tumor growth upon the cisplatin-based therapy *in vitro* and *in vivo*. Our results show that not only does *AC* protect hepatic cells but also act in synergy with cisplatin to promote lung carcinoma cell death. *AC* was further demonstrated able to reduce oxidative stress *in vitro*. Finally, we conclude that *AC* in conjunction with cisplatin inhibits growth of line-1 lung carcinoma cells and attenuates cisplatin-induced cachexia, liver damage and inflammation in mice.

## RESULTS

### *A. cinnamomea* protects hepatic cells, inhibits lung carcinoma cells and alleviates oxidative stress

**Figure 1:**
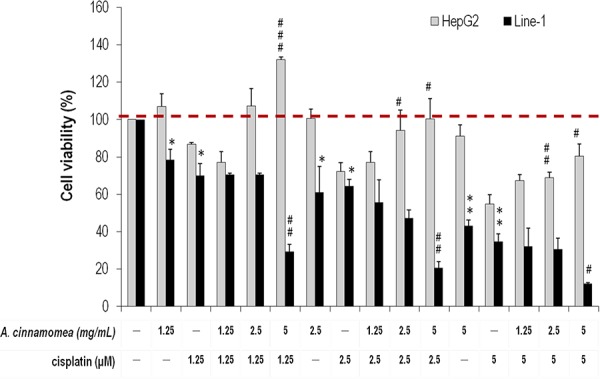
Effects of *A. cinnamomea* on cell viability in cisplatin-treated human hepatic HepG2 and mouse line-1 lung carcinoma cells Cells were incubated in culture medium containing various concentrations of cisplatin and/or *A. cinnamomea* for 48 h. After the treatment, cell viability was determined by the MTS assay. Values shown are relative to that of vehicle control, where the value of control cell viability is set to 100%, a representative of three independent experiments. Data, which are derived from at least three independent experiments, six tests for each, are presented by mean ± SEM. *P < 0.05, **P < 0.01, ***P < 0.001 versus vehicle-treated cells; ^#^*P* < 0.05, ^##^*P* < 0.01 versus cisplatin-treated cells.

To make sure whether *AC* differentially protects hepatic cells but inhibits lung carcinoma cells, we examined cell viability and survival rate in the presence of *AC* by MTS assay. For this experiment, both the mouse line-1 lung carcinoma cell line and the human hepatic HepG2 cell line were used, and results were compared to evaluate the differential cytotoxicity of *AC*. HepG2 is an immortalized human hepatoma cell line that is frequently used in hepatocyte-function-related studies [32]. Cells were treated with various concentrations of *AC* (1.25, 2.5 and 5.0 mg/mL) for 48 h, where the number of line-1 lung carcinoma cells declined significantly while that of HepG2 cells had no change (Figure 1). To know if cisplatin synergizes with *AC* to reduce cell viability, comparison assays were performed. In terms of cell viability and proliferation, HepG2 cells treated with cisplatin or/and *AC* resulted in quite distinct results as shown in Figure 1: *AC* does not inhibit the viability of HepG2 cells, while cisplatin has strong cytotoxicity to HepG2 cells in a dose-dependent manner. Surprisingly, *AC* can reverse the cisplatin-induced cell death also in a dose-dependent manner (*p* < 0.05). On the other hand, both cisplatin and *AC* do inhibit line-1 lung carcinoma cells. When both cisplatin and *AC* were administrated together, a synergistic suppression resulted (*p* < 0.05, Figure 1). Accordingly, we suggest that cisplatin/*AC* has an inhibitory effect on tumor growth (line-1 cells); *AC* has a protective effect on hepatic cells, particularly the cisplatin-induced cytotoxicity.

To conclude the above findings, we further examined the effects of *AC* on different primary cultured cells BALB/cByJ mice cell line and human A549 lung carcinoma cell line with/without addition of cisplatin. The results of BALB/cByJ mice cells indeed are consistent with those of human hepatic HepG2 cells (Supplementary Figure S2A). In contrast, the A549 cell line, which is cisplatin-resistant NSCLC cells [33, 34], displayed no observable cytotoxity under the doses of 1.25–5 μM cisplatin versus the presence/absence of *AC* (Supplementary Figure S2B). As a result, we considered that the line-1 cell line is a better testing cell model than A549 in this experiment to reflect the true anti-tumorigenesis/liver-protection effect of *AC*.

**Figure 2:**
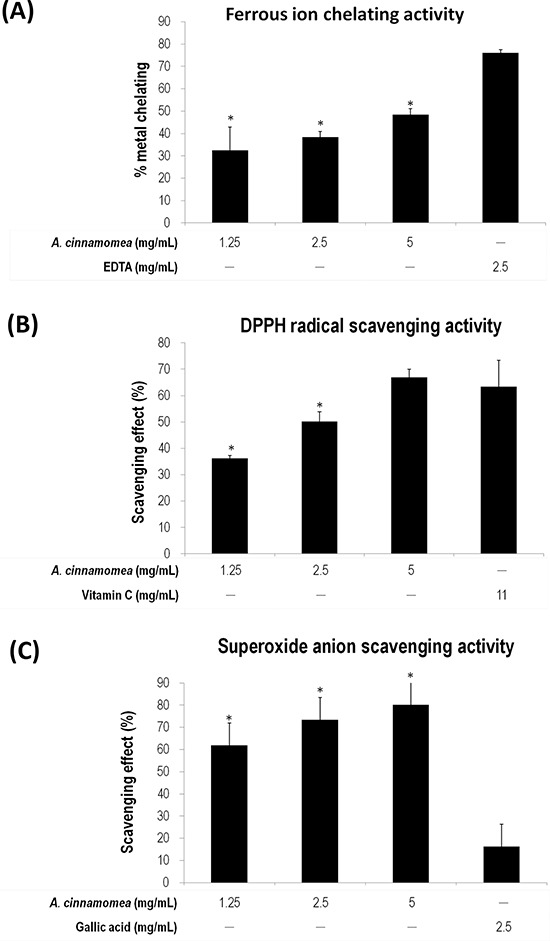
Antioxidation activity of *A. cinnamomea* The antioxidation activity for *A. cinnamomea* was determined by the ferrous ion chelating assay. **A.** the α,α-diphenil-β-picrylhydrazine (DPPH) radical scavenging assay. **B.** and the superoxide anion scavenging assay **C.** Data, which are derived from at least three independent experiments, six tests for each, are presented by mean ± SEM. *P < 0.01 indicates significant difference from the positive control.

**Figure 3:**
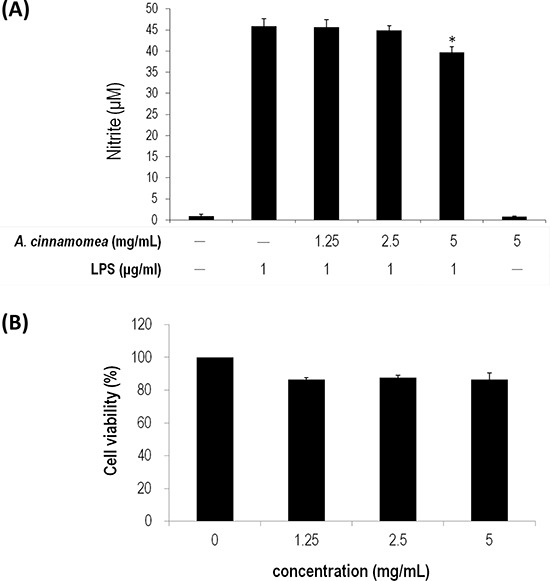
Inhibition of *A. cinnamomea* on NO production in LPS-stimulated RAW264.7 macrophages **A.** Various concentrations (1.25, 2.5 and 5 mg/mL) of *A. cinnamomea* were evaluated for NO production in LPS-stimulated RAW264.7 macrophages. **B.** Cytotoxicity of *A. cinnamomea* was determined by the MTS assay. Values shown are relative to vehicle control, where the value of control cell viability is set to 100%, a representative of three independent experiments. Data, which are derived from at least three independent experiments, six tests for each, are presented by mean ± SEM. 1 μg/mL LPS is a positive control. *P < 0.01 indicates significant difference from the positive control.

The anti-oxidative activity of *AC* was subsequently determined using ferrous ion chelating assay, DPPH radical scavenging assay and superoxide-anion scavenging assay. As shown in Figure 2A, the linear formation of ferrous and ferrozine complexes when *AC* was added indicates that *AC* can chelate irons in a dose-dependent manner, although the iron chelating activity of *AC* is not as good as that of EDTA. In Figure 2B, *AC* presents DPPH scavenging activity also in a dose-dependent manner, wherein the scavenging rates are 66.88% and 36.22% for *AC* at 5 and 1.25 mg/mL, respectively. The anti-oxidation activity of *AC* is comparable to that of Vitamin C. In Figure 2C, *AC* has a superior superoxide radicals quenching rate to gallic acid (2.5 mg/mL, 16.36%). Their quenching rates are 80.15%, 73.43%, and 61.94% in the presence of 5, 2.5, and 1.25 mg/mL of *AC*, respectively. LPS-induced RAW 264.7 cells were also subjected to NO inhibition assay, whereby nitrite accumulates. When *AC* (5 mg/mL) was included in the NO production system, the accumulation of nitrite significantly decreased (Figure 3A), where cytotoxicity was not detected (Figure 3B).

As a result, *AC* that possesses several desired biological activities is confirmed: *AC* selectively kills lung carcinoma cells without harming immortalized hepatic cells; *AC* has strong anti-inflammation and anti-oxidation activities. *AC* is a suitable agent to improve conventional chemotherapeutics in cancer treatment.

### *A. cinnamomea* in combination with cisplatin inhibits growth of line-1 lung carcinoma cells inoculated into mice

**Figure 4:**
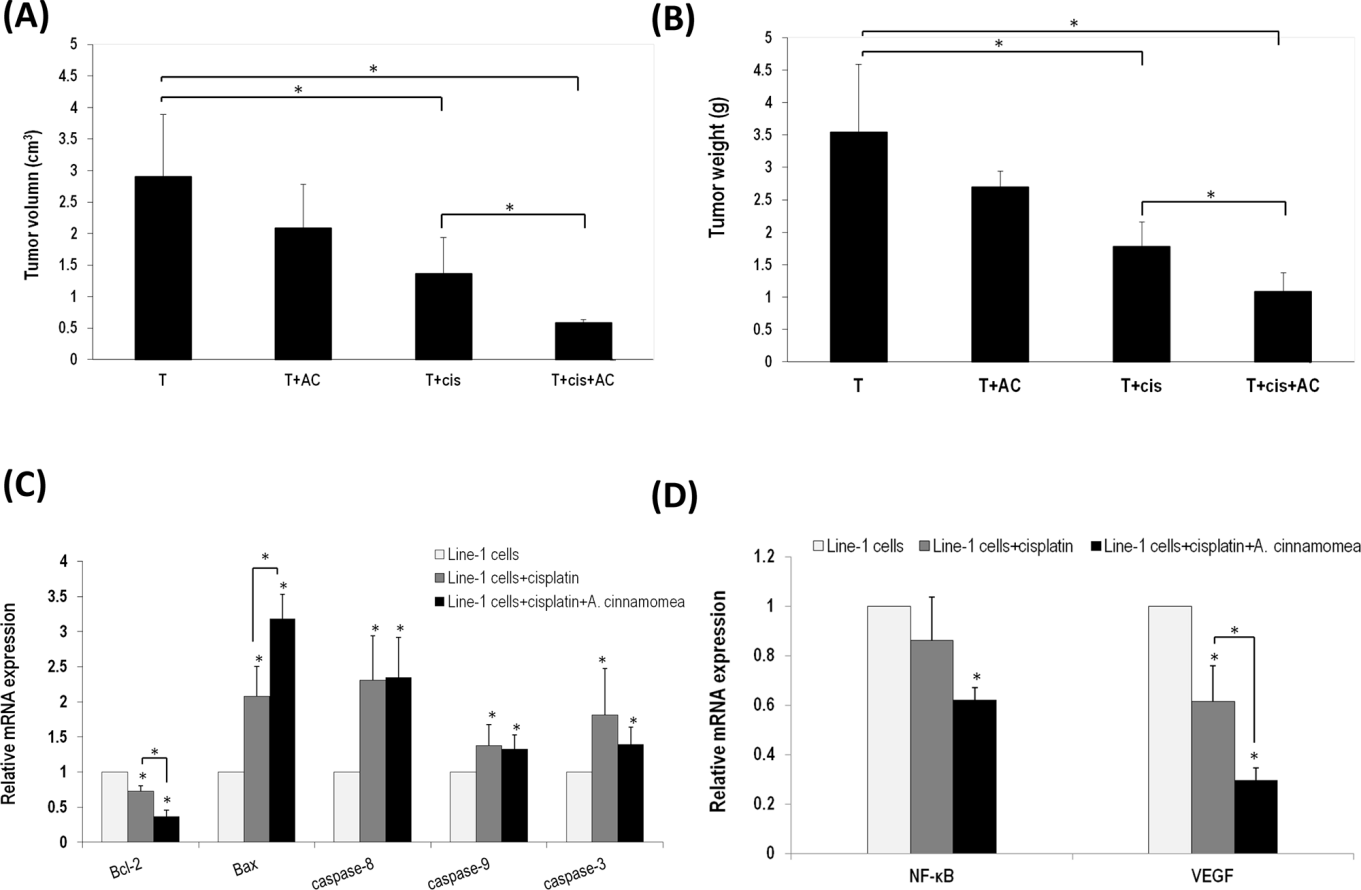
*A. cinnamomea* and cisplatin synergistically inhibit tumor growth in the line-1 xenografted mouse model **A.** Line-1 lung carcinoma cells were established in BALB/cByJ male mice (*n* = 6) by subcutaneously injecting line-1 cells (3 × 10^5^ cells) into the right thigh. Mice were orally administered with vehicle (T), 300 mg/kg *A. cinnamomea* (T+*AC*), 2.5 mg/kg cisplatin (T+cis), or a combination of *A. cinnamomea* and cisplatin (T+cis+*AC*) for 18 days. Mice were sacrificed on day 26 and then examined for their tumor volumes. Tumor volumes were calculated using the following formula: (length × width^2^)/2. **B.** Examination of tumor weights. **C.** Estimation of mRNA levels of Bacl-2, Bax, caspases 3, 8 and 9 by quantitative real-time PCR (qRT–PCR) from tumors treated with vehicle control, cisplatin, or the combination of *A. cinnamomea* and cisplatin. **D.** Quantification of mRNA levels of VEGF and NF-κB by qRT–PCR in the tumor tissues of all mice treated with vehicle control, cisplatin, or the combination of *A. cinnamomea* and cisplatin. The value of the vehicle control mRNA is set to 1. Data are expressed as the means ± SD (*n* = 6 mice per group from two independent experiments). *Significantly different from the compared group (*P* < 0.05).

Cispaltin in combination with *AC* was then assessed for their anti-tumor efficacy by examining tumor sizes, caspases activities, expressions of NF-κB and VEGF. A dose of 2.5 mg/kg cisplatin was intraperitoneally injected into BALB/cByJ mice xenografted with murine lung carcinoma line-1 cells. As shown in Figure 4A & 4B, tumor volume and tumor weight in the T+cis group decrease significantly by 52.96% and 49.80%, respectively, when compared to those of the T group. Mice that received a combination of 300 mg/kg *AC* and cisplatin reduced tumor volume and tumor weight by 79.82% and 69.36%, respectively (Figure 4A & 4B). Co-administration of *AC* and cisplatin apparently exhibits a synergistic antitumor activity.

To understand whether the synergistic effect is as result of modulating apoptotic signaling pathways, the gene expression of Bcl-2 family proteins (Bcl-2 and Bax) and caspases (3, 8 and 9) was examined by real time PCR (Figure 4C). The mRNA level of Bcl-2 decreased after treating the tumor-bearing mice with cisplatin, while that of Bax increased instead (Figure 4C). The set of testing animals co-administrated with *AC* and cisplatin showed significantly decreased anti-apoptotic Bcl-2 but increased pro-apoptotic Bax when compared with those treated with cisplatin alone. The gene expression of caspases 3, 8 and 9 increased significantly in the T+cis group. Mice that received a combination of *AC* and cisplatin did not change, but maintain an above-average gene level of caspases 3, 8 and 9. Thus, our results suggest that *AC* can maintain or enhance antitumor activity of cisplatin.

It has been well documented that cancer cells regularly express NF-κB and VEGF [35, 36]. NF-κB is a redox-sensitive transcription factor sensing oxidative stresses [37]. We found that the gene expression level of NF-κB increased in the T group, thus suggesting a high oxidative stress in tumor. The mice treated with cisplatin did not show decreased NF-κB in contrast to those co-administrated with *AC* and cisplatin (Figure 4D). Tumor inoculation would normally induce expression of angiogenic factor VEGF. The mice treated with cisplatin showed reduced VEGF; those co-administrated with *AC* and cisplatin indeed showed an enhanced effect. As a result, *AC* not only has an antineoplastic activity but also carry an antioxidant activity.

### *A. cinnamomea* attenuates cisplatin-induced liver damage and inflammation in mice

**Figure 5:**
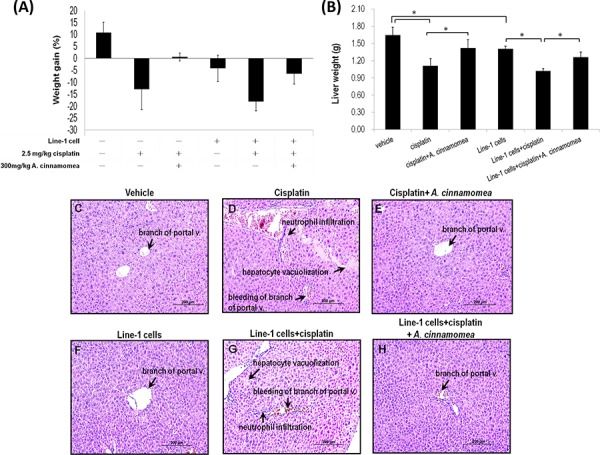
Effects of *A. cinnamomea* on cisplatin-induced body weight and liver weight loss in the line-1 xenografted mouse model At day 26, mice were sacrificed and examined for the final gains of body weights **A.** and livers weights **B.** Data are expressed as the means ± SD (*n* = 6 mice per group from two independent experiments). *Significantly different from the compared group (*P* < 0.05). **C–H.** Histopathological observation of liver. (C) vehicle group; (D) 2.5 mg/kg cisplatin treated group (cis group) showing severe bleeding, markedly inflammatory cell infiltration and vacuolar degeneration in some hepatocytes; (E) 2.5 mg/kg cisplatin *+*300 mg/kg *AC* treated group (cis+AC group) showing no pathological changes except for a few bleeding in the sinusoids; (F) line-1 cell- inoculated group (T group); (G) line-1 cell-inoculated group treated with 2.5 mg/kg cisplatin (T+cis group) showing severe bleeding, markedly inflammatory cell infiltration and vacuolar degeneration in some hepatocytes; (H) line-1 cell-inoculated group treated with 2.5 mg/kg cisplatin plus 300 mg/kg *AC* (T+cis+AC) showing no pathological changes except for a few bleeding in the branch of portal vessles. Results from 6 representative animals are shown. (magnification, all 200×).

Body weight loss has been used to index the extent of tumor-induced cachexia. As shown in Figure 5A, after a 18-day cisplatin treatment the body weight decreases 12.96% in mice (*p* < 0.05), while the body weight remains leveling off in the group receiving cis+*AC* (*p* > 0.05), suggesting that *AC* can restore the cisplatin-induced weight loss. The body-weight loss was estimated about 4.20% in the T group, as opposed to 18.12% in the T+cis group. Tumor inoculation would cause weight loss; cisplatin however aggravates the weight loss. The body weight loss in the T+cis+AC group is about 6.48%, while the cisplatin-induced body weight loss is considerably lessened with the treatment of *AC* (*p* < 0.05, Figure 5A), ascertaining that *AC* alleviates the cisplatin-induced weight loss in tumor-bearing mice.

*AC* that reduces the cisplatin-induced hepatotoxicity was further explored by measuring liver tissue weight, histopathological change and serum biochemical parameters in the mice xenografted with line-1 lung carcinoma cells. As shown in Figure 5B, the liver weights are 1.65 ± 0.14 and 1.41 ± 0.05 g in the vehicle and T groups, respectively. Tumor inoculation seems to have no effect on liver weight change, whereas cisplatin administration does cause decreases of liver weight by 32.34% and 27.45% in the T+cis and cis groups, respectively. In contrast, the decreases of liver weight are 10.24% and 13.56% in the cis+AC and T+cis+AC groups, respectively. Clearly, the mice groups receiving cisplatin-*AC* significantly show lesser liver weight loss than those receiving cisplatin only (*p* < 0.05, Figure 2B). As a result, *AC* is able to rescue the cisplatin-induced liver weight loss.

Liver damage often manifests such symptoms as necrotic hepatocytes, inflammatory cellular infiltration, cytoplasmic degeneration, and hepatocytes vacuolation. As shown in Figure 5D & 5G, the mice that received cisplatin treatment (the cis and T+cis groups) show severe bleeding, neutrophil inflitration and hepatocytes vacuolation as compare with control and T groups (Figure 5C & 5F). The mice that received both cisplatin and *AC*, however, show minor hepatic damage (Figure 5E & 5H). *AC* significantly alleviates the cisplatin-induced liver damage. Histological abnormalities also coincide with the increased levels of liver alanine aminotransferase (ALT) and aspartate aminotransferase (AST) in the cisplatin-treated mice. The mice treated with cisplatin for 18 days displayed elevated levels of serum ALT and AST in the cis and T+cis groups, indicating that cisplatin *per se* causes liver dysfunction in tumor-bearing mice (Table 1). With the *AC* treatment the serum levels of AST and ALT reversed in the cis+*AC* and T+cis+*AC* groups (Table 1). Thus, *AC* not only alleviates the cisplatin-induced liver dysfunction but also protects liver from damage.

**Table 1:** Effect of *A. cinnamomea* on cisplatin-induced hepatotoxicity

Group	AST (U/L)	ALT (U/L)
**Vehicle**	71 ± 9.91	36.67 ± 0.71
**2.5 mg/kg cisplatin**	170 ± 28.84^a^	97.33 ± 10.60^a^
**2.5 mg/kg cisplatin+300mg/kg *A. cinnamomea***	102.33 ± 24.79^b^	47 ± 12.49^b^
**Line-1 cells**	178.25 ± 47.30^a^	49.5 ± 7.94^a^
**Line-1 cells+2.5 mg/kg cisplatin**	362.50 ± 39.23^a^,^c^	74 ± 6.06^a^,^c^
**Line-1 cells+2.5 mg/kg cisplatin+300mg/kg *A. cinnamomea***	237.50 ± 27.81^a^,^d^	58.25 ± 4.27^a^,^d^

Data are expressed as means ± S.E. (*n* = 6).

a *P* < 0.05 versus the control group.

b *P* < 0.05 versus the cisplatin group.

c *P* < 0.05 versus the line-1 cell-inoculated group.

d *P* < 0.05 versus the line-1 cell-inoculated group treated with cisplatin.

**Figure 6:**
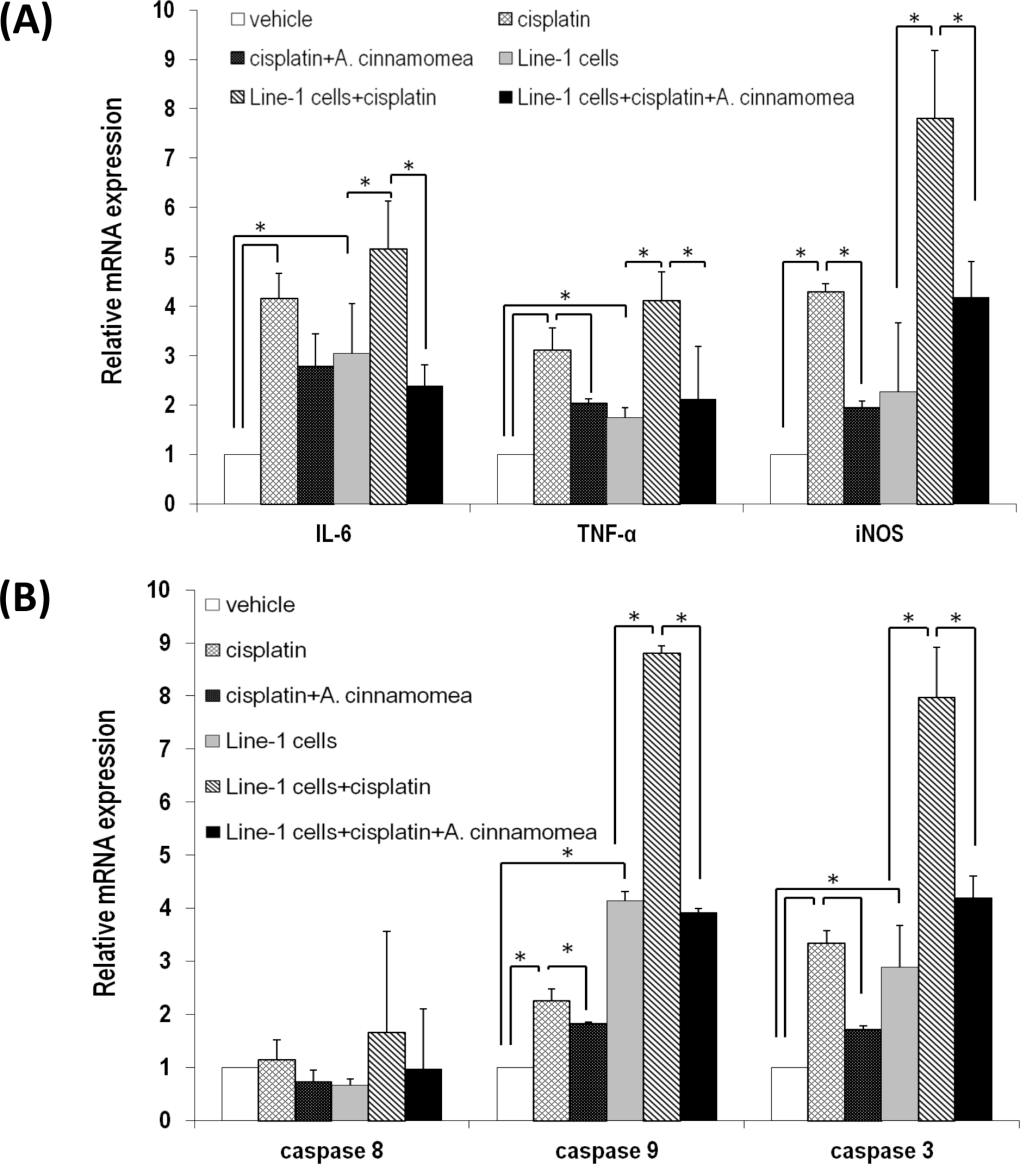
*A. cinnamomea* decreases cisplatin-induced inflammation and cell apoptosis in liver **A.** Quantification of mRNA levels of inflammatory cytokines by qRT–PCR in the liver tissues of all mice treated with vehicle control, cisplatin, or the combination of *A. cinnamomea* and cisplatin. **B.** Quantification of mRNA levels of caspases 3, 8 and 9 by qRT–PCR in the liver tissues of all mice treated with vehicle control, cisplatin, or the combination of *A. cinnamomea* and cisplatin. The value of the vehicle control mRNA is set to 1. Data are expressed as the means ± SD (*n* = 6 mice per group from two independent experiments). *Significantly different from the compared group (*P* < 0.05).

We further investigated whether *AC* has anti-inflammatory activity in liver, which indexes the extent of the cisplatin-induced tissue damage. In the cisplatin-treated mice, the level of iNOS, IL-6 and TNF-α in liver significantly increased. However, with administration of *AC* the level of these cytokines in liver decreased significantly (*p* < 0.05, Figure 6A). Expression of the apoptotic-related caspase genes 3 and 9 (except caspase 8) was increased in the cis and T+cis mice. Likewise, with the *AC* treatment the gene expression of caspases 3 and 9 kept at the basal level in the T+cis+AC mice (*p* < 0.05, Figure 6B). Cisplatin thus causes liver damage through induction of liver cell apoptosis, while *AC* can reverse the cisplatin-induced liver cell death. Taken together, *AC* is able to suppress hepatotoxicity, inflammation and apoptosis in the liver tissues of the tumor-bearing mice upon cisplatin treatment.

## DISCUSSION

To maximize tumor cytotoxicity but minimize chemotherapy-induced normal tissue damage has long been the primary task in cancer chemotherapeutic research. Many studies have reported that some natural products carry desired properties to meet this goal [38]. *AC* is one of examples able to inhibit free radical-induced oxidative stresses [26, 27] as well as to strengthen anti-tumor effects of conventional chemotherapy [28–30]. While a plethora of biological activities for *AC* were reported before [39], the hepatoprotection and lung cancer adjuvant effects, however, had never been reported.

The free radical-induced oxidative stress is now realized to play a role in the initiation and progression of tumors [40]. Researchers are looking for substances with high anti-ROS potentials, because they may stand a chance to be developed into medicines for chronic inflammatory disease and cancer. *AC* that blocks formation of oxidative stress and cell apoptosis is likely due to its high levels of flavonoids, terpinoids and polyphenolics [41]. Antroquinonol and antcin C, two active compounds in *AC*, have been shown to suppress NO and ROS in lipid peroxidation [26, 27]. In this study, we found that hot water extracts of *AC* show high level of polysaccharides, which inhibit both production of NO in the LPS-stimulated RAW264.7 macrophages and generation of superoxide and DPPH radicals.

Unlike cisplatin, of which its cytotoxicity is indiscriminable between normal and cancerous cells, *AC*, however, has no harm to normal cells. *AC* has been examined with no subchronic oral toxicity in BALB/c mice [42]; we also tested *AC* in three different dosages (350, 700 and 1400 mg/kg BW/day) in Sprague Dawley (SD) rats (10 rats/dose) for 90 consecutive days, during which we observed no mortality and detectable toxicity (data not shown). The repeat-dose toxicology for *AC* is yet to be performed, while its repeat dose in SD rats was estimated > 1,400 mg/kg BW. In this study, oral administration of *AC* significantly reduced tumor growth and promoted the cisplatin efficacy in line-1 cell-inoculated BALB/cByJ mice. *AC* also reduced inflammatory responses and formation of new blood vessels in tumor tissues, likely by down-regulating NF-κB and VEGF. Cisplatin and *AC* likely synergize in several ways through increasing pro-apoptotic Bax, decreasing anti-apoptotic Bcl-2 as well as overexpressing caspase genes 3, 8 and 9 to protect mice from the challenge of line-1 tumor cells.

The combination of natural agents and chemotherapy is getting popular in cancer therapy. One example is that curcumin, a potent radiosesitizer, supplements the expression of the radiation-induced prosurvival genes in prostate cancer patients [43]. An ideal chemo-preventing agent presumbably balances both chemosensitization and chemotherapeutic toxicity. In addition to promoting generation of ROSs, cisplatin, which non-selectively crosslinks intra- and inter-strands of DNAs [44, 45], gives rise to several severe side effects in patients. Given that cisplatin would accumulate in liver and kidney resulting strong hepatotoxicity and nephrotoxicity [46], its clinical use is thus limited. Here, we found that the *AC* co-administration not only prevents cisplatin-induced cachexia but also restores body weight/liver size to normal. *AC* was further demonstrated to alleviate the cisplatin-induced liver dysfunction, liver inflammation and hepatic apoptosis, suggesting that *AC* is promising to act as a hepaticprotective agent for patients receiving cisplatin chemotherapy [47].

In conclusion, our results evidence that *AC* is a potent adjuvant, which alleviates oxidative stress, augments antitumor efficacy of cisplatin, and protect patients from cisplatin-induced cachexia and hepatotoxicity.

## MATERIALS AND METHODS

### Cells and cell culture

Line-1 cells from the BALB/cByJ alveolar lung carcinoma (provided by Dr. John Yuhas) were adapted to tissue culture [48]. Line-1 cells were cultured in RPMI Medium 1640 (Gibco, New York, USA) supplemented with 10% fetal bovine serum (Gibco). HepG2 cells (human hepatocellular carcinoma cell line) and RAW264.7 cells (Murine macrophage cell line) were obtained from the Bioresource Collection and Research Center (Hsinchu, Taiwan). HepG2 cells were maintained in Dulbecco's modified Eagle's medium (DMEM) (Gibco), supplemented with 10% fetal bovine serum, 1% L-glutamine, 1% non-essential amino acids (NEAA) (Gibco), and 1% streptomycin-penicillin (100 IU/mL) (Hyclonr, USA). RAW264.7 cells were grown in DMEM supplemented with 10% fetal bovine serum. All cells were incubated at 37°C in a humidified atmosphere containing 5% CO_2_ for varying times.

### Preparation of *A. cinnamomea* extract

The mycelia of *AC* was provided by Chang Gung Biotechnology Corp. (Taipei, Taiwan). The *AC* extract was taken out according to a published method with slight modifications [49]. Briefly, the mycelia of *AC* were air dried and extracted with boiling water (at a ratio of 1:20, w/v) for 6 h. The extract liquid and mycelia precipitate were seperated by continuous high speed centrifugation. The suspension was filtered under suction to take out the insoluble matter. The filtrate was mixed with four volumes of ethanol (95%) and permitted to stand overnight to precipitate crude extract, in which polysaccharide is the major component. After 3890 × g centrifugation for 30 min to remove the supernatant, the crude extract referred to aqueous *AC* mycelial extract was dissolved in water and was stored at room temperature for use. The total sugar content of the *AC* extract was determined and the concentration of polysaccharide is 4.00 ± 0.80 mg/g.

### Cell viability assay

(3-(4, 5-dimethylthiazol-2-yl)-5-(3-carboxymethoxyphenyl)-2-(4-sulfophenyl)-2H-tetrazolium) (MTS) assay is a colorimetric assay based on the ability of viable cells to change from soluble yellow tetrazolium salt to blue formazan crystals. Line-1 and HepG2 cells (0.5 × 10^4^ cells/ml) were first pretreated with varying concentrations of *AC* and cisplatin for 48 h. After drug treatment, cells were washed with incubation buffer, collected by centrifugation, and then suspended in the incubation buffer, containing 0.5 mg/ml MTS for 4 h. After MTS treatment, cells were collected by centrifugation, and then suspended in DMSO for 10 min to thoroughly dissolve the dark blue crystals. The absorbency at a wavelength of 490 nm was measured by spectrophotometer. The cell viability was determined by comparing the results with the absorbency of the untreated cells.

### Nitric oxide assay

The nitric oxide assay was performed as described previously with slight modifications [50]. After pre-incubation of RAW264.7 cells (2 × 10^5^ cells/mL) with LPS (1 μg/μL) and in the presence of varying concentrations of *AC* for 18 h, the quantity of nitrite in the culture medium was measured as an indicator of NO production. Amounts of nitrite, a stable metabolite of NO, were measured using Griess reagent (1% sulphanilamide and 0.1% naphthylethylene- diamine dihydrochloride in 2.5% phosphoric acid). Briefly, 50 μL of cell culture medium was mixed with 50 μL of Griess reagent. Subsequently, the mixture was incubated at room temperature for 10 min and the absorbance at 550 nm was measured in a microplate reader. The fresh culture medium was used as a blank in every experiment. The quantity of nitrite was determined from a sodium nitrite standard curve.

### Ferrous ion chelating activity

Ferrous ion chelating activities of *AC* were determined by the method of Dinis *et al*. with slight modifications [51]. 1.7 ml distiled water, 50 μL of 0.2 mM FeCl_2_·4H_2_O, and 50 μL of 2.5 mg/mL sample solution were added into test tubes, and the mixture was left at room temperature for 1 min. 0.2 ml of 5 mM ferrozine was added, and final color was monitored at 562 nm after 10 min of incubation. In control, water was used in place of samples. The inhibition percentage of ferrozine–Fe^2+^ complex formation against blanks containing FeCl_2_ and ferrozine was calculated by the formula:

$$\%\text{ of metal chelating}=[1-({\text{A}}_{\text{s}:10}/{\text{A}}_{\text{B}:10})]\times 100$$

Where A_S:10_ is absorbance of sample and A_B:10_ is absorbance of blank at 10 min reaction time.

### DPPH radicals scavenging assay

The radical scavenging ability of *AC* was assessed by the method of Shimada *et al*. with slight modifications [52]. The radical scavenging ability of the extracts was determined at concentrations of 10 mg/mL in ethanolic DPPH solution (0.1 mM) (Sigma, Saint Louis, USA). In control, water was used in place of the sample in which the extract was prepared. Cuvettes were left in the dark at room temperature for 30 min and the resulting color was measured spectrophotometrically at 517 nm against blanks. A decreasing intensity of purple color was related to higher radical scavenging ability, which was calculated using the following equation:

$$\text{DPHH radical scavenging ability}=[1-({\text{A}}_{\text{S}:30}/{\text{A}}_{\text{B}:30})]\times 100$$

Where A_S:30_ is absorbance of the sample and A_B:30_ is absorbance of the blank at 30 min reaction time.

### Superoxide anion scavenging activity

The NADH/PMS/NBT system was used to determine the superoxide anion scavenging activity of *AC* [53]. Superoxide radicals are generated in PMS–NADH systems by oxidation of NADH and assay by the reduction of nitroblue tetrazolium (NBT) (Sigma). Briefly, 50 μL of NBT solution (300 μM in 100 mM phosphate buffer, pH 7.4) 50 μL NADH solution (936 μM in 100 mM phosphate buffer, pH 7.4) and 50 μL of sample solution (2.5 mg/ml in distilled water) were mixed. The reaction was started by adding 50 μL of phenazine methosulphate (PMS) solution (120 μM in 100 mM phosphate buffer, pH 7.4) to the mixture. The reaction mixture was incubated at room temperature for 5 min, and the absorbance at 560 nm was measured against blank samples. The decreased absorbance of the reaction mixture indicated increased superoxide anion scavenging activity. The NADH/PMS/NBT solution without sample solution was used as the control. The percent inhibition of superoxide anion generation was calculated using the following formula:

$$\text{Scavenging activity (\%)}=[1-({\text{A}}_{\text{S}:5}/{\text{A}}_{\text{B}:5})]\times 100$$

Where A_S_ is absorbance of the sample and A_B_ is absorbance of blank at 5 min reaction time.

### Mice and tumor model

Male BALB/cByJ mice (6–8 weeks) were obtained from the National Laboratory Animal Center (Taipei, Taiwan, ROC) and housed in a climate controlled room (12:12 dark-light cycle with a constant room temperature of 21 ± 1°C). Mice underwent at least 4-day adjustment to a new environment and diet before treatments were performed. Food and water were given *ad libitum*. To assess the effect of *AC* on tumor growth and cisplatin toxicity, mice were divided into groups with matched weight (Supplementary Figure S1): (1) the control group receiving normal saline (vehicle group), (2) the group treated with 2.5 mg/kg cisplatin (cis group), (3) the group treated with 2.5 mg/kg cisplatin+300 mg/kg *AC* (cis+AC group), (4) the line-1 cell-inoculated group receiving normal saline (T group), (5) the line-1 cell-inoculated group treated with 2.5 mg/kg cisplatin (T+cis group), (6) the line-1 cell-inoculated group treated with 2.5 mg/kg cisplatin plus 300 mg/kg *AC* (T+cis+AC). Tumors were induced with a subcutaneous injection of 3 × 10^5^ line-1 cells in 100 μL into the right foot dorsum of BALB/cByJ mice on Day 0. The control group was injected with 0.1 ml of a sterile saline solution. Until the 7th day after tumor inoculation (Day 7), mice were daily given *AC* (300mg/kg body weight (BW)) in 200 μL normal saline via oral gavage until sacrifice (Day 26). Cisplatin (2.5 mg/kg BW) dissolved in normal saline was intraperitoneally injected every three day on days 7 after tumor inoculation. The control group was fed orally with 0.2 ml of a sterile saline solution. Finally, 19 days later (Day 26), the mice were anesthetized and sacrificed, and the organs were removed and weighed and stored at 20°C for further analysis.

### Blood sample preparation and analyses

Blood samples (0.5 mL) were obtained immediately after killing and centrifuged immediately at 1500 × g for 10 minutes. The plasma sample was stored at 4°C within 1 h after collection and diluted 1:100 with distilled water before measurements for biochemical analysis of ALT and AST with an autoanalyser (Arkray, Japan). ALT and AST in the serum of all mice were assayed using the available kits obtained from Bio Vision Research Products (Avenue, USA). All assays were made according to the instructions of the manufacturer.

### Histopathological analysis

Tumor and liver tissues were collected from mice, washed by cold normal saline 3 times, then fixed in formalin solution 10%, processed, and embedded in a paraffin film. Sections of 5-μm thick slices of tissues were prepared. The sections were stained with H & E. Microscopic observations were carried out at 200× magnifications.

### RNA extraction and real-time PCR

Total tumor or liver tissue RNA was extracted with the Rneasy mini kit (Qiagen, Germantown, USA) and cDNA was synthesized using M-MLV reverse transcriptase (Promega, Madison, USA) and oligo-dT15- primer (Promega). Real-time PCR was performed in the Bio-Rad iCycler iQ system. Quantitative real-time PCR analysis was carried out in 25 μL reactions consisting of 12.5 μL iQ SYBR Green Supermix (Bio-Rad), 5 μL cDNA, RNase-free water, and 100 μM of each primer. Values were normalized to GAPDH mRNA amount. The oligonucleotide primers for mouse IL-6 (5′-TCCATCCAGTTGCCTTCTTG-3′ and 5′-TTTCTCATTTCCACGATTTCCC-3′), mouse TNF-α (5′-GGTGTTCATCCATTCTCTAC-3′ and 5′-CCCAGCAT CTTGTGT TTC-3′), mouse iNOS (5′-TCTCCCTTTCCT CCCTTCTTCTC-3′ and 5′-ATGGTGCC TCCTGGTGG TC-3′), mouse caspase-3 (5′-GTCTCGGTTTACCAGGA C-3′ and 5′-A CTGTCAGGGAGACTTT-3′), mouse caspase-8 (5′-ACTGTGTT TCCTACCGAG-3′ and 5′-AGC TTCTTCCGTAGTGT-3′), mouse caspase-9 (5′-CTGAGT ATTTCTCTG TGTTCCA-3′ and 5′-CATGTCACTGTTGC CC-3′), mouse Bcl-2 (5′-CCTCACCAGC CTCCTCAC-3′ and 5′-ACTACCTGCGTTCTCCTCTC-3′), mouse Bax (5′-TCTTAC AGGGTTTCATCCAG-3′ and 5′-GTCCAGT TCATCTCCAATTCG-3′), mouse VEGF (5′-GAGCAGAA GTCCCATGAAGTGAT-3′ and 5′-CAATCGGACGGCA GT AGCTT-3′), and mouse NF-κB (5′-TTACAGTAG ATGGCTACA-3′ and 5′-ATTGCT TTGTGTTGTTA-3′) were used according to previously published sequences.

### Statistical analysis

All experiments were performed at least 3 times, each time in triplicate. Data were analyzed by multivariate ANOVA test. If a significant difference was found, a least significant differences (LSD) multiple comparison test was used to identify significant groups. Statistical analyses used The Statistical Software Package for the Social Sciences, version 12.0.1 for Windows (SPSS Inc., Chicago, IL, USA). A *P* value < 0.05 was considered statistically significant.

## SUPPLEMENTARY FIGURES


