# Supplementary material for: Antrodia cinnamomea alleviates cisplatin-induced hepatotoxicity and enhances chemo-sensitivity of line-1 lung carcinoma xenografted in BALB/cByJ mice
Source: Oncotarget. 2015 Jun 27;6(28):25741–54. doi: 10.18632/oncotarget.4348 (PMC4694863; doi:10.18632/oncotarget.4348)
Supplement: Supplementary file 1 [file oncotarget-06-25741-s001.pdf]

SUPPLEMENTARY FIGURES

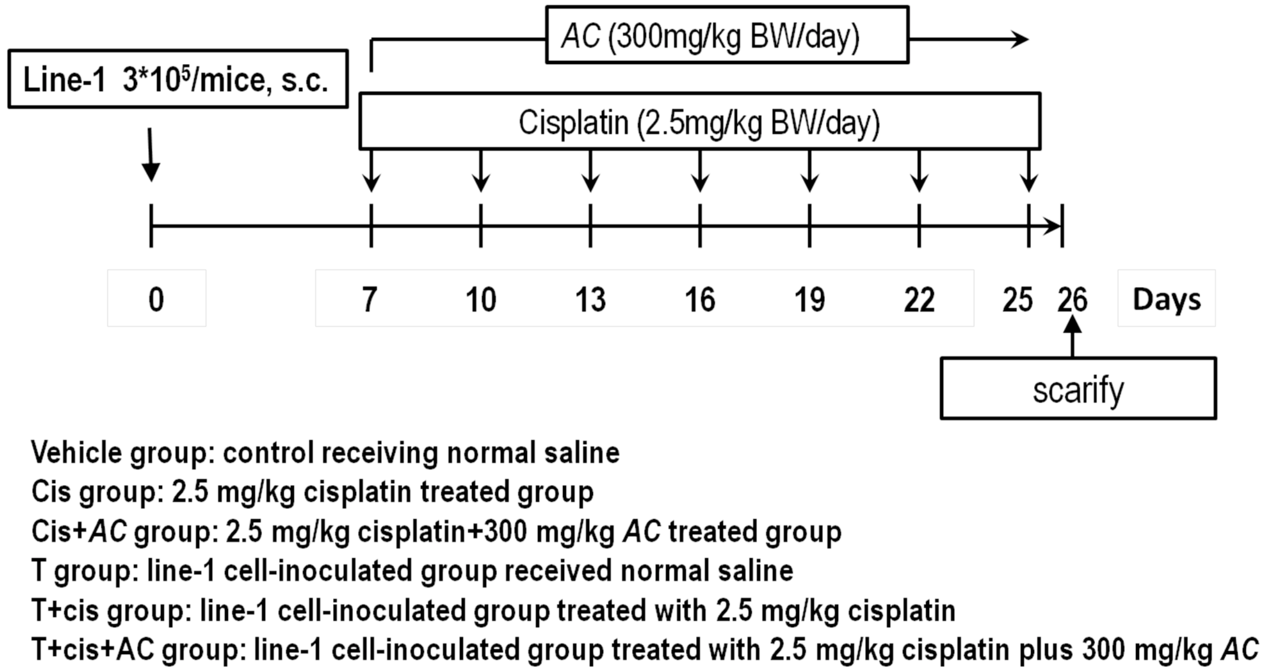

Supplementary Figure S1: The treatment protocol for tumor, cisplatin and *A. cinnamomea* in tumour-bearing mice. BALB/cByJ mice were inoculated subcutaneously (s.c.) with a homogenate of line-1 tumor cells (3×10^5 cells) on Day 0. The control group was injected with 0.1 ml of sterile saline solution. Cis-, cis+AC- and T+cis+AC-mice receiving i.p. injections of 2.5 mg/kg BW cisplatin every other days from Day 7 to Day 25. Meanwhile, cis+AC mice and T+cis+AC mice were fed orally with 300 mg/kg BW *A. cinnamomea* every day until sacrifice (Day 26). The control group was fed orally with 0.2 ml of a sterile saline solution.

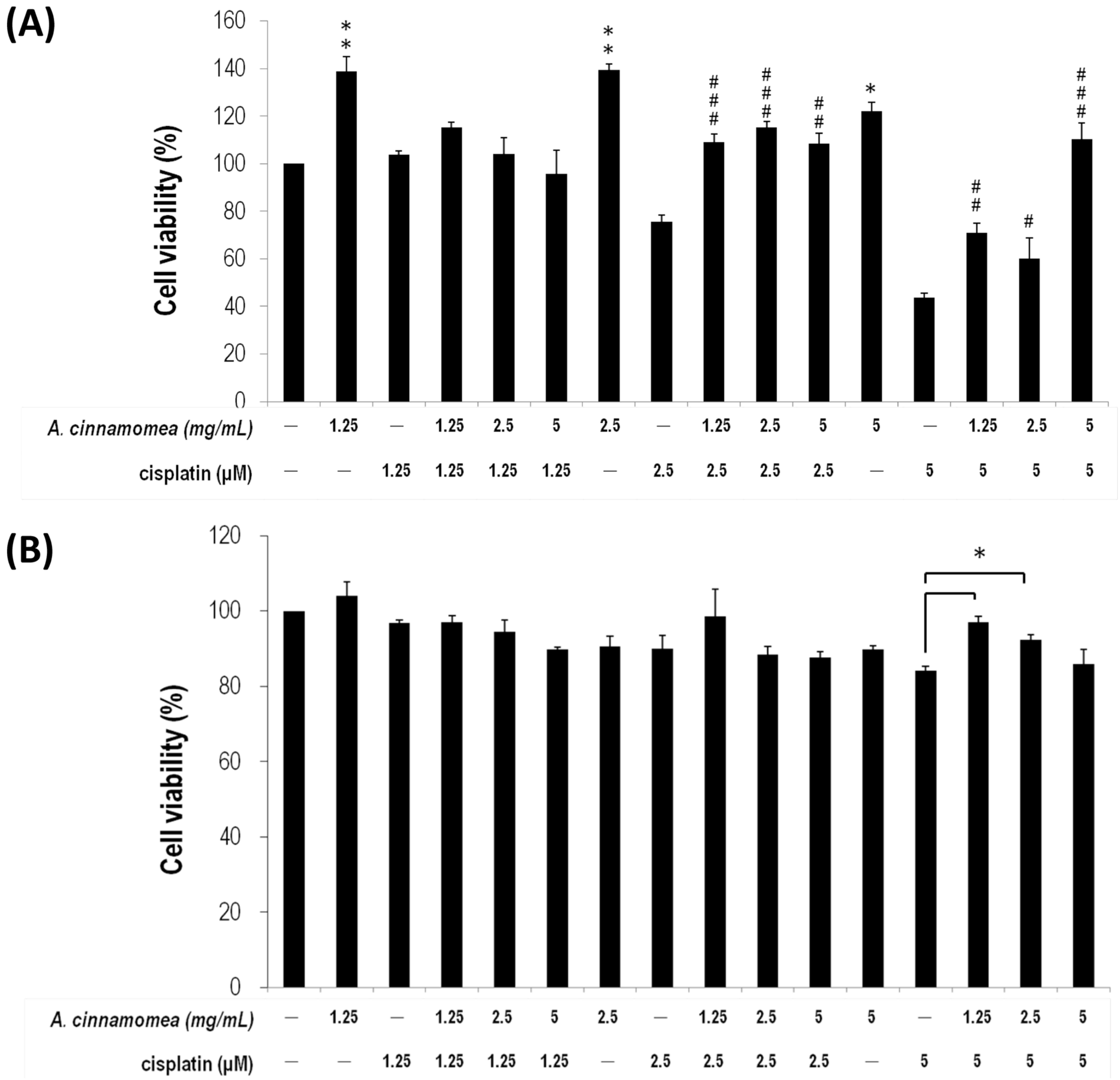

Supplementary Figure S2: Effects of *A. cinnamomea* on cell viability for cisplatin-treated primary BALB/cByJ mice liver cells and human A549 lung carcinoma cells. Primary cultured liver cells of BALB/cByJ mice **A.** and human A549 lung carcinoma cells **B.** were incubated in culture medium containing various concentrations of cisplatin and/or *A. cinnamomea* for 48 h. After treatment, cell viability was determined by the MTS assay. The values were determined relative to that of vehicle control, where the viability of control cells is set to 100% as an average of three independent experiments. All the determinations are averages of at least three independent experiments, six tests for each. Figures are presented by mean ± SEM. $^{*}P < 0.05$, $^{**}P < 0.01$, $^{***}P < 0.001$ versus vehicle-treated cells; $^{\#}P < 0.05$, $^{\#\#}P < 0.01$, $^{\#\#\#}P < 0.001$ versus cisplatin-treated cells.
